# Understanding Alignment to the Mediterranean-Style and DASH Eating Patterns and Assessing Associations with Cardiometabolic Clinical Outcomes Among Hispanic/*Latine* Adults in the United States: An NHANES Analysis

**DOI:** 10.3390/healthcare14030291

**Published:** 2026-01-23

**Authors:** Ambria Crusan, Francine Overcash

**Affiliations:** 1 Department of Nutrition and Dietetics, Henrietta Schmoll School of Health Sciences, St. Catherine University, St. Paul, MN 55105, USA; 2 Department of Food Science and Nutrition, College of Food, Agricultural and Natural Resource Sciences, University of Minnesota, St. Paul, MN 55108, USA; overc006@umn.edu

**Keywords:** cultural tailoring, evidence-based nutrition, DASH Eating Plan alignment, Mediterranean eating pattern alignment, Type 2 diabetes, hypertension

## Abstract

**Highlights:**

**What are the main findings?**

- Vast improvements are needed for both Med-style and DASH eating pattern alignment for adults of Hispanic/*Latine* origin.
- Higher alignment to DASH was associated with decreased blood pressure. Higher alignment with Med-style eating patterns was associated with a decrease in average BP and average A1c.

**What are the implications of the main findings?**

- Low adherence to both eating patterns may partly explain the ubiquitously reported poor cardiometabolic outcomes.
- Nutrition interventions supporting access and knowledge to culturally tailored eating patterns aligning with evidence-based practice are important.

**Abstract:**

**Background/Objectives**: The Mediterranean (Med)-style and Dietary Approaches to Stop Hypertension (DASH) eating patterns are evidence-based nutrition interventions given their protective effects from cardiometabolic diseases. Little is known about adherence to each eating pattern among the Hispanic/*Latine* population. The objective of this cross-sectional analysis is to assess the alignment of reported dietary intakes of Hispanic/*Latine* adults to Med-style and DASH eating patterns and associations with clinical outcomes for diabetes mellitus and cardiovascular diseases. **Methods**: A sample of 5406 Hispanic/*Latine* adults from the National Health and Nutrition Examination Survey (2007–2018) was utilized. Alignment to the Med-style and DASH eating patterns was calculated by scoring indices tailored for overconsumption in the United States. Multiple linear regression determined associations between each respective eating pattern and clinical outcomes. **Results**: Hispanic/*Latine* adults in the United States have a mean DASH score of 11.2 and a Med-style score of 8.4 (out of 100), indicating poor alignment. Adjusted regression analysis showed increased alignment of both eating patterns was associated with a decrease in average blood pressure (DASH ꞵ = −0.095, *p* = <0.0001; Med-style: ꞵ = −0.128, *p* = 0.0002). Greater adherence to a Med-style eating pattern score was also associated with improved average hemoglobin A1c (ꞵ −0.007, *p* = 0.017). Neither diet pattern score was associated with total cholesterol. **Conclusions**: Evidence of low alignment to the Med-style and DASH eating patterns among Hispanic/*Latine* populations exacerbates the need for future work to understand cultural tailoring of evidence-based eating patterns to increase adherence and support improved cardiometabolic outcomes.

## 1. Introduction

It is projected that 28% of the United States (U.S.) population will identify as Hispanic/*Latine* by 2060 [1], and diet-related chronic disease disproportionately affects those who identify as Hispanic/*Latine* in the U.S. [2,3,4]. For example, Hispanic/*Latine* adults are burdened with a 50% chance of developing Type 2 Diabetes Mellitus (DM2) compared to a 40% chance among all U.S. adults [4]. This disparity increases the need for public health solutions, often nutrition interventions targeted to reduce chronic diseases among this group, an important priority. Previous studies correlate DM2 diagnosis and hypertension (HTN), suggesting overlapping risk factors for both DM2 and other cardiovascular diseases (CVD) [5]. Moreover, social determinants of health, including inconsistent food access, limited access to health care, and decreased social support, can negatively impact one’s ability to manage chronic disease with nutrition interventions [6,7].

Both the Mediterranean (Med)-style eating pattern and the Dietary Approaches to Stop Hypertension (DASH) Eating Plan have consistently been measures of diet quality, and recommended as evidence-based nutrition interventions given their well-reported protective effects from CVD and other cardiometabolic diseases [8,9]. The 2020–2025 Dietary Guidelines for Americans’ recommendation of a Med-style eating pattern is unsurprising given the decades’ worth of compelling associations with CVD risk reduction [10]. The Med-style eating pattern, based on the Mediterranean Diet Pyramid [11], is characterized by an abundance of plant foods, lean fish, whole grains, legumes, nuts, and fresh fruits/vegetables, along with high fat from olive oil, low refined/added sugars, and wine in moderation, aligning with evidence-based recommendations for dietary management for DM2. Paralleling the Med-style eating pattern, the DASH Eating Plan is considered a nutrient-dense, balanced, and sustainable way to improve cardiometabolic markers, specifically blood pressure (BP), encouraging 4–5 servings of both fruits/vegetables, whole grains, herbs, vegetable oils, legumes, fish, poultry, and lowering of sodium [12]. The two dietary patterns differ primarily in their foundations: the Mediterranean-style eating pattern originated from traditional Mediterranean lifestyle practices that have demonstrated beneficial effects on cardiometabolic health attributed to distinctive food choices and behavioral patterns [11]. Conversely, the DASH Eating Plan was developed as a condition-specific eating pattern based on effective dietary outcomes identified in research to improve cardiovascular health [12].

Wide-spread adherence to the Med-style and DASH eating patterns in the U.S. remains elusive, according to the very limited number of ecological studies [13]. The literature on DASH Eating Plan adherence in the U.S. is outdated and limited [14,15,16], leaving a gap in the literature regarding effective dietary interventions. Unsurprisingly, even less is known about adherence to each eating pattern among racial/ethnic subgroups, such as the Hispanic/*Latine* population, in most need of the protective health effects. To date, few studies have examined clinical outcomes such as hemoglobin A1c (A1c), BP, and blood lipids, for example, based on diet interventions aligning with the Med-style or DASH eating pattern for Hispanic/*Latine* individuals living in the U.S. [17,18,19,20,21,22]. These studies are pilot studies [20,21,22] or solely focus on one of the two diet patterns as interventions [17,18,19,23]. Nonetheless, the evidence for a positive effect of both eating patterns on these subgroups is compelling and worthy of further research.

To adequately provide dietary recommendations for chronic disease management, providers require an understanding of how to adapt recommended foods and current eating patterns to meet specific cultural preferences, as well as promote access to a variety of appropriate foods [24]. More specifically, recommendations should align with culturally appropriate foods that are available and affordable to Hispanic/*Latine* populations with high prevalence of CVD, pre-diabetes (PD), and DM2. The positive effect of cultural tailoring of evidence-based dietary patterns has been demonstrated in a very limited number of trials and observational studies, mainly outside the U.S., making the case to carefully consider the eating and lifestyle habits of populations in order to achieve sustained behavior change [22,24,25,26,27,28,29]. The Hispanic/*Latine* population may greatly benefit from either diet’s established health benefits if the recommendations are culturally tailored.

Thus, the objective of this study is to assess the alignment of reported dietary intakes of Hispanic/*Latine* adults using cross-sectional data from the National Health and Nutrition Examination Survey (NHANES) in the U.S. to the Med-style and DASH eating patterns. An exploratory analysis was also conducted to understand the alignment between the Med-style and DASH eating patterns and clinical outcomes for PD/DM2 and CVD. We hypothesize that cultural tailoring of these eating patterns is needed to increase dietary alignment within the Hispanic/*Latine* population; however, we first need to understand current alignment with the diet patterns and potential associations to clinical outcomes within the Hispanic/*Latine* population subgroup.

## 2. Materials and Methods

### 2.1. Research Design

This cross-sectional sample of 5406 Hispanic/*Latine* adults ages 25–65 utilized data from NHANES 2007–2018, a survey providing an ongoing, population-based data, employing a complex, stratified, multistage probability sample design [30]. Data was obtained if participants met all eligibility criteria, including a completed demographic report, two reliable 24 h recalls, and participation in laboratory and examination assessments at the Mobile Examination Center to obtain measures for A1c, total cholesterol, weight, systolic BP, and diastolic BP, which are collected by trained professionals [31,32,33,34,35]. Adults over the age of 65 were excluded due to challenges in separating the variation in higher prevalence of diet-related chronic disease, lower dietary recall reliability, and research-supported comparability across adult life stages [36,37]. The procedures carried out to collect anthropometric, dietary, and laboratory data are detailed in the NHANES process manuals [33,34,35].

### 2.2. Measuring Instruments

Data from the U.S. Department of Agriculture’s Food Patterns Equivalents Databases (FPED), based on food pattern definitions aligned with the Dietary Guidelines for Americans [38,39], were mapped to the results for the NHANES participants’ dietary recalls. The FPED is described in detail on their resource page [39].

While multiple scoring indices have been developed to assess diet adherence to the Med-style eating pattern [40,41,42,43,44], the authors utilized a scoring index developed by Rumawas et al. [45], as it offered critical advantages over the other criterion-based scoring indices. This Med-style eating pattern score accounts for overconsumption of foods and for foods not identified as part of the Mediterranean diet, which characterizes a typical American diet [46]. Calculation of the Med-style eating pattern score is cited in our previous work [47]. To parallel the scoring created for the Med-style eating pattern, which allows for comparative analysis and quantifies adherence to the DASH Eating Plan [12], the current study utilized the same scoring index model to create a DASH score. The developed DASH score accounts for overconsumption of food groups and foods not identified as part of the DASH Eating Plan.

The DASH scoring index is based on recommended intakes of eight food groups and two key nutrient components from the DASH Eating Plan [12,48,49]. Each individual score for the aforementioned ten components is aligned to a continuous scale ranging from 0 to 10, with 10 meaning the recommended intake amount was met per the DASH Eating Plan. First, the average intake by day for each DASH Eating Plan food component was calculated by averaging the FPED data across the two 24 h recalls collected in the NHANES. Next, the component’s average intake by day was multiplied by a designated number of points based on recommended intake amounts in the DASH Eating Plan. The ten DASH Eating Plan food components were assigned a specific number of points, so that a maximum score of 10 would be attained if the recommended number of servings per day—or, in the current study’s case, the average intake per day—were met. The components were assigned points as follows: whole grains, 3.33 points; fruits, 2.5 points; vegetables, 2.50 points; dairy, 5.00 points; fish or seafood, 3.33 points; fish and other seafood, poultry and egg, 3.33 points; legumes and nuts, 2.50 points; oils, 6.67 points. Nutrient values for sodium and potassium, as well as sweets and sugar-sweetened beverages, were computed incrementally per the amount over the recommendation. For example, for every 80 mg of sodium over the DASH recommendation of 1500 mg, the score was reduced by 1 point. For potassium, for every 470 mg over 4700 mg, 1 point was reduced. Sweets and sugar-sweetened beverages reduced the score by 1 point for every serving over an average of 0.75 servings/day.

Overconsumption of foods that did not align with the DASH Eating Plan was factored in via percentage of the pre-component score exceeding the recommended amount, with points subtracted from the component score for numbers of servings over the recommendation [45,47]. The theoretical maximum sum was determined by adding all individual component scores and standardizing them to a 0–100 scale (dividing the total sum by 100). This scoring index is tailored for non-DASH populations by using a weighting factor (0–1) to account for typical DASH foods compared to non-DASH foods. Each food consumed was systematically categorized by FPED category as a DASH food group or a non-DASH food group [12,39], from which a proportion of the total energy of non-DASH foods to the total energy of DASH foods was derived. Categories included as DASH foods included total fruit, total vegetables, total dairy, poultry, seafood (both low and high O-3), soy, cooked and dry beans, nuts and seeds, oils, eggs, and whole grains. Alternatively, food categories that were considered non-DASH aligned were: meat, organ meat, and cured meat, refined grains, added sugars, drinks containing alcohol (more than one serving), and solid fats. The theoretical maximum sum was subsequently multiplied by the weighting factor as the final calculation step. A higher total DASH score indicates higher adherence to the DASH Eating Plan (range, 1–100).

Clinical outcome measures were categorized for analysis according to current clinical diagnostic levels set by their respective associations. Average BP was categorized as Normal: Systolic is <120 AND diastolic < 80 mmHg, Elevated:120–129 mmHg AND <80 mmHg, Stage 1 HTN: 130–139 mmHg/80–89 mmHg, Stage 2 HTN: 140–179 mmHg/90–11 mmHg, and Crisis: >180/120 mmHg, and total cholesterol was categorized as Normal: <200 mg/dL, Borderline High: 200–239 mg/dL, and High: >239 mg/dL according to guidance from the American College of Cardiology and American Heart Association [50,51]. A1c was categorized as Normal: <5.7%, PD: 5.7–6.4%, DM2: 6.5–10.9%, and Uncontrolled DM2: >11% according to the American Diabetes Association [52].

### 2.3. Statistical Analyses

Statistical analyses used various survey procedures in SAS v.4.0 (SAS Institute Inc., Cary, NC, USA) to account for stratification, clustering, and weighting (NHANES supplied) inherent in the complex, large study population of NHANES. Descriptive analysis included calculating frequencies/percentages of descriptive characteristics and means and 95% confidence intervals of DASH and Med-style eating pattern scores using PROC SURVEYFREQ and PROC SURVEYMEANS, respectively. Multiple linear regression analyses, adjusted for age, sex, and education using PROC SURVEYREG, determined the impact of each eating pattern score as a continuous variable on the three clinical outcome variables (average BP, A1c, total cholesterol), respectively. To further elucidate the linear regression modeling results, each clinical outcome was categorized into diagnostic categories and weighted means, and standard deviations by level were calculated. Adjusted logistic regression modeling was performed to calculate pairwise comparisons of the eating pattern scores between the different levels of each clinical variable.

## 3. Results

Of the 5406 individuals who self-reported as Hispanic/*Latine*, specifically Mexican in NHANES, the majority reported they had U.S. citizenship status (58%) with an equal distribution of males and females (Table 1). The largest proportion of participants were in the low-income poverty-to-income ratio group (46%) and had some college/associates degree (47%). Over half of the study population fell within normal levels for all three clinical measures, respectively: average BP (54%), total cholesterol (57%), and A1c (66%). In contrast, the least number of participants met the criteria for healthy body mass index (BMI) (18%), while the largest percentage of participants fell in the obese category (44%). The study population’s unadjusted mean (95% CI) DASH and Med-style scores were 11.2 (10.9, 11.6) and 8.4 (8.1, 8.6), respectively.

The adjusted regression analysis showed alignment to both DASH and Med-style eating patterns and was associated with a decrease in average BP, separately (Table 2). Average BP decreased by 0.095 mmHg with every one-point increase in DASH score (*p* < 0.0001). For every one-point increase in Med-style eating pattern, average BP decreased by 0.127 mmHg (*p* = 0.0002). Greater alignment to a Med-style eating pattern score was also associated with improved average A1c. For every one-point increase in Med-style eating pattern score, there was a 0.007 unit decrease in A1c (*p* = 0.019). DASH was not associated with improved average A1c, and neither diet pattern score was associated with total cholesterol.

To add context to the calculated regression coefficients, pairwise comparisons of means for both eating pattern scores by diagnostic levels of average BP and A1c were calculated (Table 3 and Table 4). Levels of the HTN diagnostic levels (Normal, Elevated, Stage 1 HTN, Stage 2 HTN, Crisis) differ by DASH and Med-style pattern scores similarly, as demonstrated by the same pattern of significant pairwise differences (Table 3). Those who met criteria for “Normal HTN” for both eating pattern scores have significantly different mean scores from those with “Stage 1 HTN”, “Stage 2 HTN”, and “Crisis”. The magnitude of point differences between levels is similar, all less than ~0.3 points for each pairwise comparison of both scores separately. Unsurprisingly, the largest difference in magnitude, slightly over three points, was seen between “Normal BP” and “Crisis” for both scores. Interestingly, our data suggests participants may have greater alignment to the DASH eating pattern than to the Med-style eating pattern, given that both scores are on a scale of 1 to 100. The remaining pairwise differences were the same for both scores: participants with “Elevated HTN” differed only from those with “Crisis” BP levels; participants with “Stage 1 HTN” and “Stage 2 HTN” differed from those in “Crisis” BP levels. The only significant pairwise difference in mean Med-style eating pattern score was between those with Normal A1c and those with DM2 (~0.6 of a point) (Table 4).

## 4. Discussion

A robust body of research supports the effectiveness of the Med-style or DASH eating patterns for the prevention and management of cardiometabolic diseases, such as DM2 and HTN, as well as other cardiometabolic risk factors [53,54,55,56,57]. However, a very limited number of these studies examine these associations among racial/ethnic subgroups in most need of the protective health effects. To our knowledge, the current study is the first to show associations between both DASH and Med-style eating pattern scores and clinical outcomes in population-wide data of Hispanic/*Latine* individuals in the U.S. Moreover, the Med-style and DASH eating patterns have been predominantly researched in association with people diagnosed with CVD or DM2; their effectiveness is less certain in the management of other cardiometabolic diseases among more specific cultural groups that share food patterns and preferences. More research to examine whether greater alignment would benefit cultural subgroups.

Findings from the current study add to the body of evidence that vast improvements are needed for both Med-style and DASH eating pattern alignment for adults of Hispanic/*Latine* origin [17,18,23]. Our main findings indicate that Hispanic/*Latine* adults in the U.S. show poor alignment to both eating patterns, with a mean DASH score of 11.2 (out of 100) and a mean Med-style score of 8.4 (out of 100). Because these eating patterns are renowned dietary interventions for improving chronic conditions, the low adherence found among our Hispanic/*Latine* study population may partly explain the ubiquitously reported poor cardiometabolic outcomes. A previous study from the seminal project, the Hispanic Community Health Study/Study of Latinos (HCHS/SOL), the largest population-based cohort study of the Hispanic/*Latine* subcultures (16,415 participants across four urban U.S. cities), found similarly low Med-style eating pattern adherence in the U.S. [17]. This warrants further investigation into the potential effects of significantly improving alignment on cardiometabolic outcomes, especially given the paradigm shift to studying diet patterns on the whole rather than focusing on the improvement of one to two nutrients and/or food groups [58].

A number of studies from the HCHS/SOL cohort exploring food patterns in Hispanic/*Latine* communities found that higher adherence to healthy dietary patterns was significantly associated with lower risk of incident CVD after six years [17]. Specifically, each one-standard-deviation increase in the diet pattern score corresponded to approximately a 20–26% lower CVD risk (RR ~0.80 and RR ~0.74, respectively) [17]. This suggests that clinically meaningful improvements in cardiometabolic markers may require substantial shifts in dietary pattern alignment over time. Our exploratory analysis on cardiometabolic outcomes conducted in the regression models showed that a one-point increase for either diet pattern’s score did not translate to a clinically meaningful change for any of the three clinical outcomes. However, with the large margin of misalignment in both diet pattern scores, a clinically significant increase of one point may also be the wrong focus. Instead of trying to improve the score nominally, larger increases in scores, for example, a 50-point increase in diet pattern score, would equate to a more clinically meaningful change in A1c (−0.35) with the Med-style eating pattern. The same idea would be applicable in BP, as a 50-point increase in score would decrease blood pressure by 5 mmHg with the DASH Eating Plan and by ~7 mmHg with the Med-style eating pattern. For example, findings from the Multicenter HCHS/SOL showed that increased adherence to the DASH Eating Plan among 10,741 participants was associated with lower systolic and diastolic BP outcomes, with each 10-unit increase in the DASH score, systolic blood pressure declined by ~0.36 mmHg and diastolic blood pressure by ~0.62 mmHg [23].

Cultural tailoring of evidence-based nutrition interventions may support sustainable dietary behavior change and serve as a cost-effective solution for greater community-wide impact. One study by Minari et al. [59] provided personalized diet planning aligned to a combination of the Med-style and DASH eating patterns, adapted to Brazilian foods, and considered socioeconomic status. Their findings showed nominal changes in BP would occur when following this diet pattern, whereas A1c changes would be more impactful. Similar to our findings showing that there was no change in total cholesterol based on greater alignment to the DASH or Med-Style eating patterns, the authors did not find clinically meaningful changes in total cholesterol with their adapted combination diet pattern. However, the authors’ prior work [47] underscores potential effects for greater alignment to the Med-style eating pattern with small diet swaps over 2 days, which could increase scores by 4 points. If diet pattern recommendations were met with increased compliance to guidelines, a clinically meaningful change could be anticipated. Additional strategies for adaptations may be needed to increase alignment, as traditional foods have been reported as the most difficult to change upon acculturation to a new country [60]. Cultural adaptations, including the use of staple foods, ingredients, and spices that align with either pattern, may increase adherence from a hedonic perspective that in turn increases sustainability [28].

While cultural tailoring is proving to be an effective method to apply evidence-based nutrition interventions to cultural subgroups, we recognize that improved alignment to either eating pattern may be impeded by certain social determinants of health. For example, low socioeconomic status, food security, and barriers to access and availability of nutrient-dense foods are challenges that can be consistently experienced by Hispanic/*Latine* populations in the U.S. [61,62,63]. Seafood and olive oil, arguably the quintessential components of the Med-Style eating pattern, are found to be more expensive and less consumed, especially among low-income Americans [64,65]. However, the Boston Puerto Rican Study identified inexpensive cultural staples that align with the principles of the Med-Style eating pattern [27], demonstrating that alignment to these diet patterns is plausible. Previous research also shows the adaptability of the DASH Eating Plan using traditional Hispanic/*Latine* foods [22,66], especially fruits and vegetables, versus replacing cultural foods with options that are not part of typical eating patterns. Our findings showing DASH eating pattern scores at baseline were higher than the Med-Style eating pattern score (11.2 vs. 8.4, respectively) in this study population suggest there is already a greater alignment to the DASH Eating Plan in comparison to the Med-Style eating pattern. Moreover, unlike the Med-style eating pattern, the DASH eating pattern is not defined by the inclusion of expensive foods. As such, choosing the optimal eating pattern may entail more than just improving diagnostic outcomes for these cultural subgroups.

An unexpected result from building our final adjusted regression model was that education was a significant factor influencing the effect of the DASH eating pattern on A1c; once education was added, significance was lost, which was not the case for the Med-style eating pattern. This finding may suggest level of education may play a more impactful role in explaining the association between alignment to DASH and A1c for Hispanic/*Latine* adults than it does in measuring the same association between Med-style eating pattern and A1c. This finding also suggests the importance of sound nutrition education in attaining greater alignment with the DASH eating pattern if improvement in A1c is of primary interest. In order to improve alignment with the DASH eating pattern, one requires more concrete knowledge of nutrition topics. For example, understanding how to achieve the “prescribed” amount of sodium or the appropriate portion sizes to meet the goals of 4–5 servings of fruits and vegetables per day. Previous research supports that food and nutrition literacy supports a stronger understanding of portion sizes and composition of foods, which is associated with higher educational attainment [67,68]. Therefore, if the DASH eating pattern is more suited to desired outcomes, it is imperative to consider educational needs to ensure adequate adherence to the diet pattern.

The strength of this work is that this is the first epidemiological study to assess alignment to both the Med-style and DASH eating patterns among Hispanic/*Latine* groups in the U.S. This study is also the first, to our knowledge, that utilizes a DASH eating pattern alignment score that accounts for overconsumption, a common pattern found in U.S. diets. With a large sample size provided by the NHANES, this is a nationally representative dataset that provides the ability to disaggregate the data and better understand disparities. However, a primary limitation of the study is that dietary intakes are self-reported by the people who participated in the NHANES study, which can be subject to recall and social desirability biases. Other limitations are that the NHANES data provides a cross-sectional opportunity for analysis. Therefore, reverse causation and the natural variability in diet patterns over time cannot be assessed. Due to the nature of the percentage of people with crisis-level blood pressure readings and uncontrolled DM, our sample sizes for those groups were small. Moreover, we accounted for demographic factors that were present in our preliminary analyses, and there is still the potential for effects of other confounding factors missed in the analysis. For example, we did not account for medications taken due to the significant number of missing responses. Future research is necessary to address gaps in this work to effectively explore the feasibility of culturally aligned Med-style and DASH diet patterns for the Hispanic/*Latine* population in the U.S.

Future work is needed to better understand cultural tailoring of advice to align with evidence-based eating patterns such as Med-style and DASH. Adherence to eating patterns is higher when the intervention is aligned with the participant’s typical and cultural eating patterns [61,69,70]. This study builds upon prior work [59], highlighting that the Med-style and DASH eating patterns are not only culturally adaptable but also promote a non-restrictive approach to include important cultural foods. To date, pragmatic and cultural perspectives held by Hispanic/*Latine* adults supporting either eating pattern are very limited. A better understanding is needed regarding culturally relevant characteristics to ensure the intervention’s intended population not only meets its behavior change objectives, but also sustains them.

## 5. Conclusions

Now more than ever, the paradigm is shifting towards preventative health. Evidence for the effectiveness of the Med-style and DASH eating patterns is robust and continues to grow in specificity and depth. Therefore, nutrition interventions supporting access and knowledge to culturally tailored eating patterns aligning with these evidence-based interventions are even more important. There are clear diet-related health disparities in the Hispanic/*Latine* population, requiring mitigation via effective public health solutions. The current study clearly demonstrates low alignment to the Med-style and DASH eating patterns among Hispanic/Latine populations, highlighting the need to understand cultural tailoring of evidence-based eating patterns to increase adherence and that may result in sustainable yet impactful improvements in cardiometabolic outcomes.

## Figures and Tables

**Table 1:** Descriptive characteristics and mean DASH and Med-style eating pattern scores of study population (*n* = 5406).

Characteristic	Frequency ^1^	Percent
Gender		
Male	2520	50.1
Female	2886	49.9
Age		
25–39 years	1901	47.0
40–49 years	1265	26.2
≥50 years	2240	26.8
Poverty–Income Ratio (PIR)		
Low (<1.3) ^2^	2577	45.6
Middle (1.3–3.49)	1878	35.2
High (≥3.5)	951	19.3
Education		
High school/GED or less	2356	38.7
Some college/Associate degree	2369	46.9
College graduate or higher	676	14.4
Citizenship status		
Citizen by birth or naturalization	3135	57.7
Not a citizen of the United States	2235	41.7
Average Blood Pressure		
Normal	2691	54.1
Elevated	927	16.7
Stage 1 HTN	968	17.7
Stage 2 HTN	707	11.0
Crisis	42	0.56
Total Cholesterol		
Normal (<200 mg/dL)	2991	57.3
Border High (200–239 mg/dL)	1639	29.2
High (>239 mg/dL)	776	13.5
Glycohemoglobin A1c		
Normal: <5.7%	3239	66.2
Pre-DM: 5.7–6.4%	1486	24.3
DM2: 6.5–10.9%	605	8.3
Uncontrolled: >11%	76	1.2
Body Mass Index Status		
Underweight	22	0.4
Healthy	950	18.0
Overweight	2004	37.2
Obese	2395	44.3
Eating Pattern Score	Mean Score	95% Confidence Interval
DASH	11.2	10.9, 11.6
Med-style	8.4	8.1, 8.6

^1^ missing data accounts for subtotals less than n = 5406. ^2^ 1.3 = 130% of the federal poverty level.

**Table 2:** Adjusted ^1^ linear regression model examining associations between DASH and Med-style eating pattern scores and cardiometabolic outcomes.

	DASH Eating Pattern Score		Med-Style Eating Pattern Score	
Cardiometabolic Measure	Beta (Std Err)	Regression Coefficient *p*-Value	Beta (Std Err)	Regression Coefficient *p*-Value
Average blood pressure	−0.095 (0.020)	<0.0001	−0.128 (0.033)	0.0002
Total cholesterol	−0.151 (0.079)	0.06	0.049 (0.111)	0.660
Glycohemoglobin A1c	−0.004 (0.002)	0.057	−0.007 (0.003)	0.017

^1^ all models adjusted for sex, age, education, and U.S. citizenship status.

**Table 3:** Adjusted^1^ logistic regression model of DASH and Med-style eating pattern scores by diagnostic categories of average blood pressure.

	DASH Eating Pattern Score	Med-Style Eating Pattern Score		
Average BP Category	Least Square Mean ^1^ (Std Err)	Least Square Mean^1^ (Std Err)	DASH Score Pairwise Differences ^2–6^	Med-Style Score Pairwise Difference ^2–6^
Normal HTN	11.973 (0.168)	8.873 (0.132)	c, d, e	c, d, e
Elevated HTN	11.664 (0.280)	8.750 (0.206)	e	e
Stage 1 HTN	11.071 (0.247)	8.438 (0.198)	a, e	a, e
Stage 2 HTN	11.010 (0.260)	8.221 (0.229)	a, e	a, e
Crisis	8.832 (0.929)	5.656 (0.545)	a, b, c, d	a, b, c, d

^1^ Least square mean models adjusted for sex, age, education, and U.S. citizenship status. ^2^ a = pairwise difference with Normal, *p* < 0.05. ^3^ b = pairwise difference with Elevated, *p* < 0.05. ^4^ c = pairwise difference with Stage 1 HTN, *p* < 0.05. ^5^ d = pairwise difference with Stage 2 HTN, *p* < 0.05. ^6^ e = pairwise difference with Crisis, *p* < 0.05.

**Table 4:** Adjusted^1^ logistic regression models of DASH and Med-style eating pattern scores by diagnostic categories of hemoglobin A1c.

	DASH Eating Pattern Score	Med-Style Eating Pattern Score		
Average DM (A1c) Category	Least Square Mean ^1^ (Std Err)	Least Square Mean ^1^ (Std Err)	DASH Score Pairwise Differences ^2,3^	Med-Style Score Pairwise Difference ^2,3^
Normal: <5.7%	11.617 (0.201)	8.615 (0.154)	n/a	f ^2^
Pre-DM: 5.7–6.4%	11.199 (0.219)	8.360 (0.183)	n/a	n/a
DM2: 6.5–10.9%	10.870 (0.369)	8.073 (0.236)	n/a	g ^3^
Uncontrolled: >11%	11.443(0.780)	7.87 (0.502)	n/a	n/a

^1^ Least square mean models adjusted for sex, age, education, and U.S. citizenship status. ^2^ f = pairwise difference with DM2, ^3^ g= pairwise difference with Normal, *p* < 0.05. n/a = not statistically significant.

## Data Availability

The study used all publicly available data sets: NHANES (CDC), and the USDA’s Food Patterns Equivalents Database and Food.

## Abbreviations

The following abbreviations are used in this manuscript:

A1c	hemoglobin A1c
BMI	body mass index
BP	blood pressure
CVD	cardiovascular diseases
DASH	Dietary Approaches to Stop Hypertension
DM2	type 2 diabetes mellitus
FPED	Food Patterns Equivalents Databases
HCHS/SOL	Hispanic Community Health Study/Study of Latinos
HTN	hypertension
Med-style	Mediterranean-style
NHANES	National Health and Nutrition Examination Survey
PD	pre-diabetes
U.S.	United States
